# Proteome-wide analysis of lysine acetylation in the plant pathogen *Botrytis cinerea*

**DOI:** 10.1038/srep29313

**Published:** 2016-07-06

**Authors:** Binna Lv, Qianqian Yang, Delong Li, Wenxing Liang, Limin Song

**Affiliations:** 1 The Key Laboratory of Integrated Crop Pest Management of Shandong Province, College of Agronomy and Plant Protection, Qingdao Agricultural University, Qingdao 266109, China

## Abstract

Lysine acetylation is a dynamic and reversible post-translational modification that plays an important role in diverse cellular processes. *Botrytis cinerea* is the most thoroughly studied necrotrophic species due to its broad host range and huge economic impact. However, to date, little is known about the functions of lysine acetylation in this plant pathogen. In this study, we determined the lysine acetylome of *B. cinerea* through the combination of affinity enrichment and high-resolution LC-MS/MS analysis. Overall, 1582 lysine acetylation sites in 954 proteins were identified. Bioinformatics analysis shows that the acetylated proteins are involved in diverse biological functions and show multiple cellular localizations. Several particular amino acids preferred near acetylation sites, including K^ac^Y, K^ac^H, K^ac^***R, K^ac^F, FK^ac^ and K^ac^***K, were identified in this organism. Protein interaction network analysis demonstrates that a variety of interactions are modulated by protein acetylation. Interestingly, 6 proteins involved in virulence of *B. cinerea*, including 3 key components of the high-osmolarity glycerol pathway, were found to be acetylated, suggesting that lysine acetylation plays regulatory roles in pathogenesis. These data provides the first comprehensive view of the acetylome of *B. cinerea* and serves as a rich resource for functional analysis of lysine acetylation in this plant pathogen.

Lysine acetylation is one of the most common post-translational modifications (PTMs) to proteins in both eukaryotes and prokaryotes. Lysine acetylation, including histone acetylation and non-nuclear protein acetylation, is a dynamic and reversible process. The acetylation status of histones has been extensively studied in the regulation of gene transcription via the modulation of nucleosomal DNA package^1^,^2^,^3^. Furthermore, the discovery of lysine acetylation in non-histone proteins has greatly expanded our understanding of functions of this modification^4^. To date, protein acetylation has been found to occur in almost every compartments of a cell, such as the cytoplasm and mitochondria^5^,^6^. It plays important roles in many cellular physiological processes, including cell-cycle regulation and apoptosis, cell morphology^6^, metabolic pathways^7^,^8^,^9^,^10^, protein interactions^10^ and enzymatic activity^9^,^11^. In addition, acetylation has also been implicated in regulating the beneficial effects of calorie restriction, a low nutrient diet without starvation and aging^12^,^13^. Therefore, lysine acetylation is believed to be a major signaling modality because of its ubiquitous occurrence and diverse biological functions^14^,^15^.

Due to the important role of lysine acetylation in the regulation of protein functions and cellular processes, acetylomes have been determined in many species. Advances in mass spectrometry (MS)-based proteomics have greatly enabled the post-translational modification to be studied on a proteomic scale^13^,^16^,^17^,^18^. In recent years, the acetylomes of many eukaryotes^5^,^6^,^13^,^18^,^19^,^20^,^21^,^22^,^23^,^24^ and prokaryotes^7^,^25^,^26^,^27^,^28^,^29^,^30^,^31^ have been identified using proteomic methods. These acetylome studies have generated large datasets of PTM sites, which demonstrate the diverse cellular functions of lysine acetylation in these species. The acetylome identification on a large scale greatly increases the knowledge of lysine acetylated proteins and expands the global view of their functional landscape.

Although lysine acetylation is evolutionarily conserved from prokaryotes to eukaryotes^6^, little is known about the function of this modification in filamentous fungi. *Botrytis cinerea*, a necrotrophic plant pathogen, can cause grey mold on more than 200 plant species^32^. The financial impact of grey mold is enormous because *B. cinerea* causes both pre- and post-harvest losses^33^. Therefore, it is of considerable interest to examine the regulatory function of lysine acetylation in this important plant pathogen. In this study, we performed the first large-scale analysis of lysine acetylated proteins in *B. cinerea*. We identified 1582 lysine acetylation sites in 954 proteins which are involved in a variety of biological functions and are localized to multiple cellular compartments. Several particular amino acids preferred near acetylation sites similar to those found in other species were also identified in this organism. Interestingly, 6 proteins involved in virulence of *B. cinerea* were identified to be acetylated proteins, including 3 key components of high-osmolarity glycerol (HOG) pathway. This work provides the first extensive dataset on lysine acetylation in *B. cinerea*.

## Results

### Proteome-wide analysis of lysine acetylation sites and proteins in *B. cinerea*

**Figure 1:**
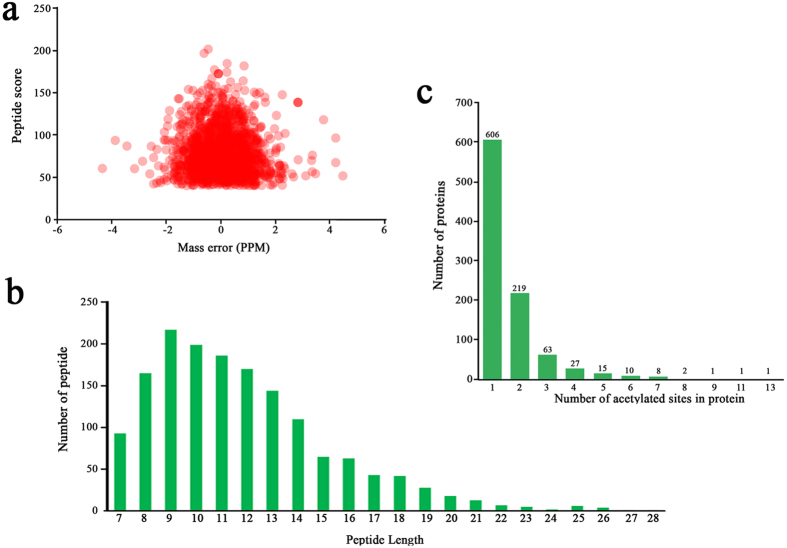
Proteome-wide identification of lysine acetylation sites in *B. cinerea.* (**a)** Mass error distribution of all identified peptides. **(b)** Peptide length distribution. **(c)** Distribution of acetylated proteins based on their number of acetylation peptides.

The whole genome of *B. cinerea* was previously sequenced^34^, which facilitates systematic analysis of lysine acetylated proteins in this species. To determine the protein acetylome in *B. cinerea*, a proteomic method was applied. The MS data validation was shown in Fig. 1. The distribution of mass error was near zero and most of them were less than 6 PPM which means the mass accuracy of the MS data fits the requirement (Fig. 1a). In addition, the length of most peptides was distributed between 7 and 19, which agrees with the property of tryptic peptides (Fig. 1b). Therefore, sample preparation reaches the standard. Altogether, 1582 acetylation sites in 954 proteins were identified (Supplementary Table S1), which account for 5.82% (954/16389) of the total proteins in *B. cinerea*. The acetylated proteins contained different numbers of acetylation sites from 1 to 13 (Fig. 1c). As shown in Fig. 1, 63.5% of the acetylated proteins contain only one acetylation sites, and the percentage of proteins with two, three, four or more modification sites are 23%, 6.6% and 6.9%, respectively. The mass spectrometry proteomics data have been deposited to the ProteomeXchange Consortium via the PRIDE partner repository with the dataset identifier PXD004031.

### Characterization of the lysine acetylome of *B. cinerea*

**Figure 2:**
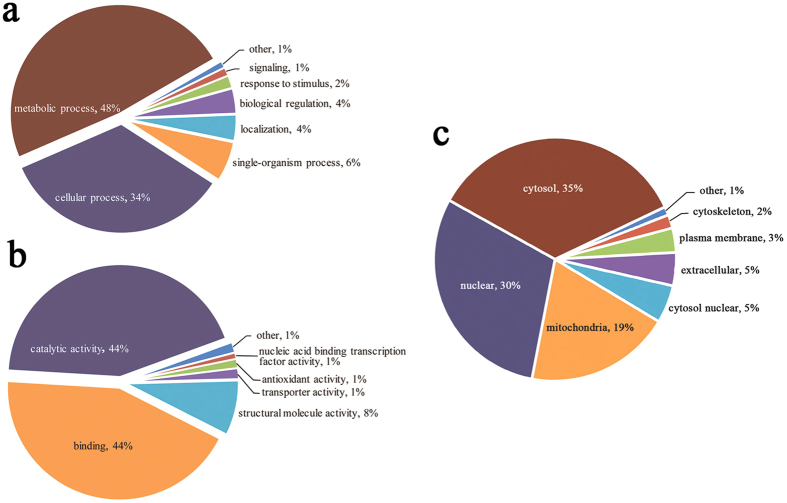
Gene Ontology functional classification of the identified acetylated proteins. (**a)** Classification of the acetylated proteins based on biological process. **(b)** Classification of the acetylated proteins based on molecular function. **(c)** Subcellular localization of the acetylated proteins.

To understand the lysine acetylome in *B. cinerea*, we investigated the GO functional classification of all the identified proteins based on their biological process and molecular function (Fig. 2a,b, Supplementary Table S2, Supplementary Table S3). According to GO annotation information, we calculated the number of identified proteins in each GO term. The classification results for biological process and molecular function showed that enzymes involved in metabolism formed the largest protein group of acetylated proteins, which account for 48 and 44% of the identified proteins, respectively (Fig. 2a,b, Supplementary Table S2, Supplementary Table S3). This is consistent with previous studies that a large proportion of lysine acetylated proteins are categorized as metabolic proteins in other organisms^7^,^25^,^26^,^27^,^28^,^29^. Another large acetylated protein group determined by their molecular function classification is composed of binding proteins, which account for 44% of all the acetyl proteins (Fig. 2b). The second largest group in terms of biological process is proteins associated with cellular processes, which account for 34% of all the identified proteins (Fig. 2a). These results indicate that the acetylated proteins, with diversified molecular functions, are involved in a variety of biological processes in *B. cinerea*.

The subcellular localization of the acetylated proteins was also analyzed, and the results show that they are mainly distributed in the cytosol (35%), nucleus (30%) and mitochondria (19%) (Fig. 2c). The overall trend in the subcellular localization of the acetylated proteins is similar to previous results obtained in *Arabidopsis* and silkworm^22^,^24^.

**Figure 3:**
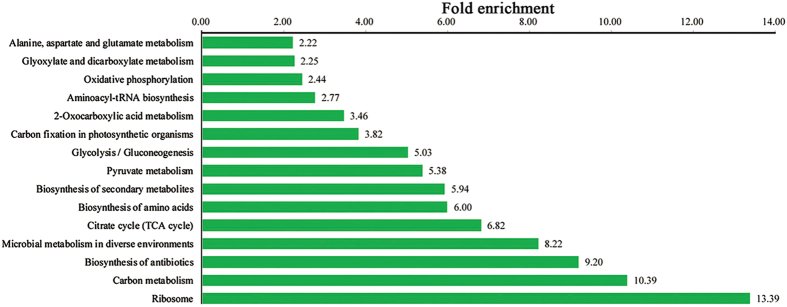
KEGG pathway-based enrichment analysis of identified proteins.

To better understand the characteristics of the acetylated proteins in *B. cinerea*, we conducted functional enrichment of GO (biological process, molecular function, and cellular component categories), KEGG pathway and protein domain analyses. As determined through the GO and KEGG analyses, proteins involved in ribosome and carbon metabolism were most enriched (Fig. 3, Supplementary Fig. S1, Supplementary Tables S2–S5). In agreement with these observations, the domain enrichment analysis showed that proteins with translation protein SH3-like domain and ribosomal protein L2 domain 2 have a higher tendency to be acetylated (Supplementary Fig. S2, Supplementary Table S6). These results indicate that proteins associated with ribosome are most likely acetylated in *B. cinerea*. Moreover, the enrichment of pathways in *B. cinerea* termed microbial metabolism in diverse environments and biosynthesis of secondary metabolites (Fig. 3, Supplementary Table S5) suggests that lysine acetylation may contribute to its pathogenesis and survival under stress conditions.

Based on these findings, we conclude that acetylated proteins have a broad range of biological functions in *B. cinerea*.

### Analysis of acetylated lysine motifs in *B. cinerea*

**Figure 4:**
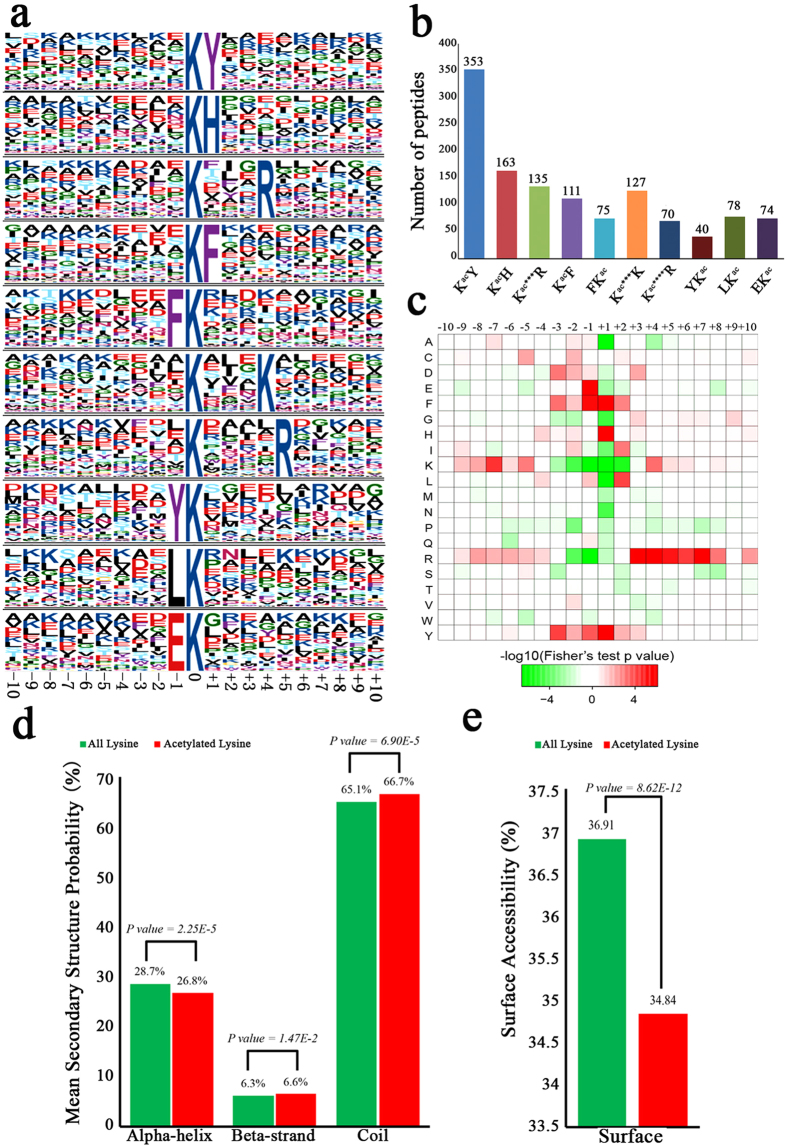
Properties of the acetylated peptides. (**a**) Acetylation motifs and conservation of acetylation sites. (**b**) Number of identified modification sites in each acetylated protein. **(c)** Heat map of the amino acid compositions of the acetylation sites. **(d)** Probabilities of lysine acetylation in different protein secondary structures (alpha-helix, beta-strand and coil). **(e)** Predicted surface accessibility of acetylated sites.

In order to understand the properties of acetylation sites, the occupancy frequency of amino acids surrounding the identified modification sites was examined. As shown in Fig. 4, using motif-x program, 1226 peptides (accounted for 77.5% of total peptides identified) were found to include the amino acid sequence from the −10 to the +10 positions surrounding the acetylated lysine. These sequences were matched to a total of ten definitively particular amino acids preferred near acetylation sites, namely, K^ac^Y, K^ac^H, K^ac^***R, K^ac^F, FK^ac^, K^ac^***K, K^ac^****R, YK^ac^, LK^ac^ and EK^ac^ (Fig. 4a), which exhibit different abundances (Fig. 4b) (Kac indicates the acetylated lysine and * indicates a random amino acid residue). Motifs K^ac^Y, K^ac^H and K^ac^F were particularly conserved as peptides with these motifs account for approximately 40% of all the identified peptides. Enrichment of residues tyrosine (Y), histidine (H), arginine (R), phenylalanine (F) and lysine (K) were found downstream of the acetylated lysine, whereas tyrosine (Y), glutamic acid (E), leucine (L) and phenylalanine (F) were found upstream of the modification site. For these motifs, the enrichment of aromatic amino acids (Y and F) and a positively charged amino acid (H) was observed in the +1 position. These data suggest that aromatic and positively charged amino acids may be functionally important for acetylation. Moreover, the heat map of the amino acid compositions surrounding the acetylation sites showed that the frequency of K and R in positions −2 to −1 in the motifs is the lowest, whereas the frequency of F, H and Y is the highest (Fig. 4c). Therefore, proteins with such motifs are preferred substrates of lysine acetytransferases in the cell. Among the ten motifs identified in *B. cinerea*, some were also observed in other eukaryotic and prokaryotic species^6^,^20^,^21^,^24^,^26^,^28^,^35^, supporting the notion that lysine acetylation is a highly conserved post-translational modification.

To elucidate the relationship between acetylation and protein structure in *B. cinerea*, the secondary structures of acetylated proteins was investigated in this study. As shown in Fig. 4d, the majority of acetylation sites was located on coils (66.7%) rather than on alpha-helix (26.8%) and beta-strand (6.6%) in *B. cinerea* (Fig. 4d), which was similar to previous studies^24^. Although the distribution pattern of acetylated lysine and all lysine is similar (Fig. 4d), there is still tendency of acetylation in *B. cinerea* based on the P values shown in Fig. 4d. Surface accessibility of acetylated lysine sites was also evaluated. The results showed that approximately 34.8% of acetylated lysine sites were exposed to protein surface, compared with 36.9% of all lysine residues (Fig. 4e). Therefore, lysine acetylation can only slightly affect the surface property of proteins in *B. cinerea*.

### Analysis of protein interaction networks of acetylated proteins in *B. cinerea*

**Figure 5:**
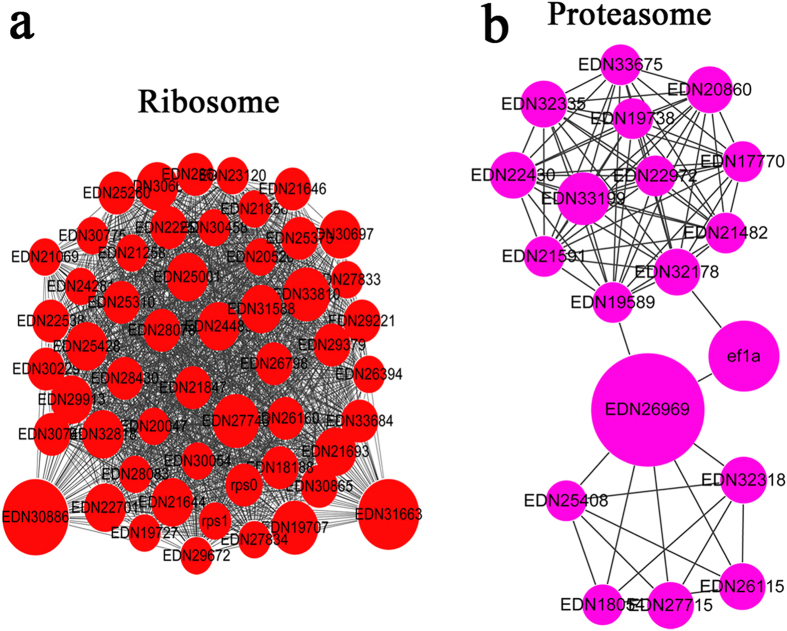
Interaction networks of acetylated proteins in *B. cinerea*. **(a)** Interaction network of acetylated proteins associated with ribosome. **(b)** Interaction network of acetylated proteins associated with proteasome.

To further understand the cellular processes regulated by acetylation in *B. cinerea*, the protein interaction network was established. 954 acetylated proteins were mapped to the protein network database, and the global network graph of these interactions was shown in Supplementary Fig. S3 and Supplementary Table S7. Twenty-four highly interconnected clusters of acetylated proteins were retrieved using the Cytoscape software (Fig. 5, Supplementary Table S7). The results showed that acetylated proteins involved in a certain pathway comprise a dense protein interaction network. The subnetwork graphs of acetylated proteins in the top two clusters (ribosome and proteasome) were shown in Fig. 5a,b. Other subnetworks for metabolic pathways, oxidative phosphorylation, microbial metabolism in diverse environments, aminoacyl-tRNA biosynthesis, endoplasmic reticulum and RNA transport were shown in Supplementary Fig. S4 and Supplementary Table S7. These subnetworks also have a relatively high density. These results indicate that the physiological interactions among acetylated protein complexes are likely to contribute to their cooperation in *B. cinerea*.

### Analysis of acetylated proteins involved in virulence of *B. cinerea*

In this study, six proteins involved in the virulence of *B. cinerea* were found to be acetylated, including three key components of high-osmolarity glycerol (HOG) pathway: BcSak1, Hpt1 and BOS1 (Table 1). In addition, two chitin synthase, Bcchs2 (Gene Bank accession no. XP_001557191.1) and BcchsV (Gene Bank accession no. XP_001545514) and one polyketide synthase (Gene Bank accession no. XP_001545729.1) which is one of the key enzyme of biosynthesis of the toxic secondary metabolites were also found to be modified by acetyl groups. These results suggest that acetylated proteins may play a role in virulence of *B. cinerea*.

## Discussion

Although lysine acetylation is a widespread and highly conserved post-translational modification in both eukaryotes and prokaryotes with diverse biological functions^6^, little is known about the function of this modification in filamentous fungi. In this study, we determined the lysine acetylation sites in the proteome of *B. cinerea* using the combination of affinity enrichment and high-resolution LC-MS/MS analysis. A total of 954 acetylated proteins with 1582 lysine acetylation sites were identified in *B. cinerea*, which greatly expands the catalog of acetylated proteins in filamentous fungi. Intensive bioinformatics analysis showed that the acetylated proteins are widely distributed and participate in diverse biological processes. The identification of several particular amino acids preferred near acetylation sites suggests substrate preference of lysine acetylation in *B. cinerea*. Furthermore, protein interaction network analysis demonstrated that a wide range of interactions are modulated by protein acetylation. These data represents the first comprehensive view of the acetylome in *B. cinerea*.

As a necrotrophic fungus, *B. cinerea* has the ability to infect many different plant species and tissues under various environmental conditions. Many research efforts have been made in the understanding of its infection strategies^36^. In this study, six proteins involved in the virulence of *B. cinerea* were found to be acetylated, including three key components of high-osmolarity glycerol (HOG) pathway: BcSak1, Hpt1 and BOS1 (Table 1). These results indicate that acetylated proteins may play a role in virulence of *B. cinerea*. In fungi, the HOG pathway is involved in the sensitivity and resistance to fungicides, phenylpyrrole and dicarboximide (e.g., iprodione), and it could be activated by a variety of external stimuli, including fungicides, osmotic stress and hormones^37^,^38^,^39^,^40^. In *B. cinerea*, several putative elements of the pathway, including the osmosensor histidine kinase BOS1, the histidine phosphotransfer protein Hpt1, the response regulator BcRrg1, the mitogen-activated protein kinase kinase kinase (MAPKKK) BcOs4, the MAPKK BcOs5 and the MAPK BcSak1 have been identified. To date, all these core elements except for Hpt1, which is essential for *B. cinerea*, have been characterized. BOS1, BcOs4, BcOs5 and BcSak1 are required for full virulence in *B. cinerea*^41^,^42^,^43^,^44^,^45^. In addition, further studies revealed that the majority of Sak1-regulated genes are unrelated to stress responses, but rather, involved in general metabolic functions^46^. In support of our observations, p38, an ortholog of the yeast HOG1p MAP kinase (BcSak1 in this study), was found to be acetylated in human. Acetylation of the lysine residue, K53, of p38 protein, regulates the kinase activity of this enzyme^47^. Although MAPK cascades consist of protein kinases that are sequentially activated by phosphorylation, in this study, the discovery that three key components of HOG pathway were acetylated offers new insights into the understanding of MAP Kinase signal transduction pathways. Additional experiments will be needed to conclusively prove these findings.

Since the penetration structures of *B. cinerea* need a stable cell wall, several chitin synthase isoenzymes were shown to be important for its pathogenicity^48^,^49^. In this study, two chitin synthase, Bcchs2 (Gene Bank accession no. XP_001557191.1) and BcchsV (Gene Bank accession no. XP_001545514), were found to be modified by acetyl groups. These results suggest that acetylation plays an important role in cell wall integrity and virulence of *B. cinerea*. Moreover, the toxic secondary metabolites play a critical role in host killing. The enzyme responsible for the committed biosynthetic step is often referred to as the “key” enzyme. Forty three genes in *B. cinerea* were predicted to encode key enzymes: 16 type I iterative Polyketide Synthase, nine Non-Ribosomal Peptide Synthetase, five PKS/NRPS, one DiMethylAllylTryptophan Synthase, one type III PKS (chalcone synthase), and 11 terpene synthases^36^. Of all the key enzymes, one polyketide synthase (Gene Bank accession no. XP_001545729.1) was identified as acetylated protein, which means lysine acetylation might play a role in secondary metabolism.

In conclusion, we determined the lysine acetylome of *B. cinerea* for the first time. Our findings showed that acetylated proteins are associated with various biological functions and are distributed in a number of cellular compartments of *B. cinerea*. Most importantly, several proteins involved in virulence of *B. cinerea* were found to be modified by acetyl groups. Overall, our results indicate that the regulatory scope of lysine acetylation is broad in *B. cinerea*.

## Methods

### Strains and culture

Strain B05.10 of *B. cinerea Pers. Fr*. [*B. fuckeliana* (de Bary) Whetzel] is an isolate from *Vitis vinifera* (Germany) and is widely used as a standard reference strain^50^. The strain was cultured on PDA (200 g potato, 20 g glucose, 20 g agar, and 1 L water) plates. The mycelia were harvested after the strain was grown in potato dextrose broth at 25 °C with shaking at 180 rpm for 2 days.

### Protein extraction and digestion

Sample was first ground in liquid nitrogen and sonicated three times on ice using a high intensity ultrasonic processor (Scientz) in lysis buffer (8 M urea, 1% Triton−100, 65 mM DTT and 0.1% Protease Inhibitor Cocktail IV, 3 μm Trichostatin A, 50 mM nicotinamide, 2 mM EDTA). The debris was removed by centrifugation at 20,000 g at 4 °C for 10 min, and then the protein was precipitated with 15% cold TCA for 2 h at 4 °C. After centrifugation at 4 °C for 10 min, the supernatant was discarded. After three times wash with cold acetone, the protein was redissolved in buffer (8 M urea, 100 mM NH_4_CO_3_, pH 8.0) and the protein concentration was determined with 2-D Quant kit (GE Healthcare) according to the manufacturer’s instructions.

For digestion, the protein solution was reduced with 10 mM DTT for 1 h at 37 °C and then alkylated with 20 mM iodoacetamide for 45 min at room temperature in darkness. For trypsin digestion, the protein sample was diluted by adding 100 mM NH_4_CO_3_ to urea concentration less than 2M. Finally, trypsin (Promega) was added at 1:50 trypsin-to-protein mass ratio for the first digestion overnight and 1:100 trypsin-to-protein mass ratio for a second 4 h-digestion.

### HPLC fractionation

The sample was fractionated into fractions by high pH reverse-phase HPLC using Agilent 300Extend C18 column (5 μm particles, 4.6 mm ID, 250 mm length). Briefly, peptides were first separated with a gradient of 2% to 60% acetonitrile in 10 mM ammonium bicarbonate pH 10 over 80 min into 80 fractions. Then, the peptides were combined into 8 fractions and dried by vacuum centrifuging.

### Affinity enrichment of lysine acetylated peptides

For acetylated peptides enrichment, 10 mg peptides were separated into 8 fraction using HPLC fractionation. Thus, for each fraction, 1.25 mg tryptic peptides dissolved in NETN buffer (100 mM NaCl, 1 mM EDTA, 50 mM Tris-HCl, 0.5% NP-40, pH 8.0) were incubated with pre-washed acetyllysine antibody agarose beads (PTM Biolabs) at 4 °C overnight with gentle shaking. The beads were washed four times with NETN buffer and twice with ddH_2_O. The bound peptides were eluted from the beads with 0.1% trifluoroacetic acid. The eluted fractions were combined and vacuum-dried. The resulting peptides were cleaned with C18 ZipTips (Millipore) according to the manufacturer’s instructions.

### Proteomic analysis by LC-MS/MS

Peptides were dissolved in 0.1% formic acid (FA) and directly loaded onto a reversed-phase pre-column (Acclaim PepMap 100, Thermo Scientific). Peptide separation was performed using a reversed-phase analytical column (Acclaim PepMap RSLC, 50 μm × 15 cm, 2 μm, 100 Å, Thermo Scientific). The gradient was comprised of an increase from 7% to 20% solvent B (0.1% FA in 98% acetonitrile) for 20 min, 20% to 35% for 8 min and climbing to 80% in 2 min then holding at 80% for the last 5 min, all at a constant flow rate of 300 nl/min on an EASY-nLC 1000 UPLC system. The composition of the A mobile phase is 0.1% formic acid (FA). The resulting peptides were analyzed by Q Exactive^TM^ Plus hybrid quadrupole-Orbitrap mass spectrometer (ThermoFisher Scientific).

The peptides were subjected to NSI source followed by tandem mass spectrometry (MS/MS) in Q Exactive^TM^ Plus (Thermo) coupled online to the UPLC. For MS scans, the m/z scan range was 350 to 1800, and ion charge was set from +2 to +5. Intact peptides were detected in the Orbitrap at a resolution of 70,000. Peptides were selected for MS/MS using NCE setting as 33; ion fragments were detected in the Orbitrap at a resolution of 17,500. A data-dependent procedure that alternated between one MS scan followed by 16 MS/MS scans was applied for the top 16 precursor ions above a threshold ion count of 1.5E4 in the MS survey scan with 10.0 s dynamic exclusion. The electrospray voltage applied was 2.0 kV. Automatic gain control was used to prevent overfilling of the orbitrap; 5E4 ions were accumulated for generation of MS/MS spectra. For MS scans, the m/z scan range was 350 to 1800. Fixed first mass was set as 100 m/z.

### Database search

The resulting MS/MS data was processed using MaxQuant with integrated Andromeda search engine (v.1.4.1.2). Tandem mass spectra were searched against *UniProt_Botrytis cinerea* B05.10 (16,581 sequences) database concatenated with reverse decoy database. Trypsin/P was specified as cleavage enzyme allowing up to 4 missing cleavages, 5 modifications per peptide and 5 charges. Mass error was set to 10 ppm for precursor ions and 0.02 Da for fragment ions. Carbamidomethylation on Cys was specified as fixed modification and oxidation on Met, acetylation on lysine and acetylation on protein N-terminal were specified as variable modifications. False discovery rate thresholds for protein, peptide and modification site were specified at 1%. Minimum peptide length was set at 7. All the other parameters in MaxQuant were set to default values. The site localization probability was set as >0.75. The other parameters were listed below: (1) variable modification: acetyl (protein N-term), Oxidation (Met); (2) fixed modification: carbamidomethyl (Cys); (3) digestion mode: trypisin/P; (4) max missed cleavages: 2; (5) first search PPM: 20; (6) main search PPM: 5; (7) max number of modifications per peptide: 5; (8) min peptide length: 7; (9) min score for modified peptides: 40.

### Bioinformatics analyses

Gene Ontology (GO) annotation proteome was derived from the UniProt-GOA database (www. http://www.ebi.ac.uk/GOA/). The identified acetylated proteins were grouped into biological process and molecular function classes based on GO terms using Blast2GO software^51^. In order to predict subcellular localization, a subcellular localization predication soft Wolfpsort was used in this research^52^. GO, KEGG pathway and protein domain enrichment were performed using the DAVID bioinformatics resources 6.7^53^. Correction for multiple hypothesis testing was carried out using standard false discovery rate control methods. The GO, KEGG pathway and domain with a corrected p-value < 0.05 were considered significant. Identified proteins domain functional description were annotated by InterProScan (a sequence analysis application) based on protein sequence alignment method using the InterPro domain database^54^. Soft motif-x was used to analyze the model of sequences constituted with amino acids in specific positions of modifier-21-mers (10 amino acids upstream and downstream of the site) in all protein sequences, and a position-specific heat map was generated by plotting the log10 of the ratio using the “heatmap.2” function from the “gplots” R-package^55^. Secondary structures were predicted using NetSurfP^56^. The protein-protein interaction networks for the identified acetylated proteins were analyzed by Cytoscape software using interaction data are from the PPI database^57^.

## Additional Information

**How to cite this article**: Lv, B. *et al*. Proteome-wide analysis of lysine acetylation in the plant pathogen *Botrytis cinerea*. *Sci. Rep.* **6**, 29313; doi: 10.1038/srep29313 (2016).

## Supplementary Material

Supplementary Figures

Supplementary Table S1

Supplementary Table S2

Supplementary Table S3

Supplementary Table S4

Supplementary Table S5

Supplementary Table S6

Supplementary Table S7

## Figures and Tables

**Table 1:** Acetylated proteins involved in virulence of *B. cinerea.*

Protein	GeneBank accession no.	Annotation	Positions
BOS1	XP_001561289.1	two-component osmosensing histidine kinase BOS1	201, 821
HPT1	XP_001549504.1	the phosphotransfer protein, similar to *S. cerevisiae* YPD1	161
BcSak1	XP_001558337.1	Mitogen-Activated Protein Kinases, Serine/Threonine Kinases	130
Bcchs2	XP_001557191.1	chitin synthase 2	166
CHSV	XP_001545514.1	chitin synthase of classes V	1118
PKS	XP_001545729.1	polyketide synthase	1047
