# Supplementary material for: Proteome-wide analysis of lysine acetylation in the plant pathogen Botrytis cinerea
Source: Sci Rep. 2016 Jul 6;6:29313. doi: 10.1038/srep29313 (PMC4933888; doi:10.1038/srep29313)

**Proteome-wide analysis of lysine acetylation in the plant pathogen *Botrytis cinerea***

Binna Lv^†^, Qianqian Yang^†^, Delong Li, Wenxing Liang^*^, Limin Song^*^

The Key Laboratory of Integrated Crop Pest Management of Shandong Province, College of Agronomy and Plant Protection, Qingdao Agricultural University, Qingdao 266109, China. †These authors contributed equally to this work. *Correspondence and requests for materials should be addressed to L.S. (andrewhjliang@163.com) or W.L. (wliang1@qau.edu.cn).

**Figure S1. GO enrichment analyses based on biological process, molecular function and cellular component categories.**


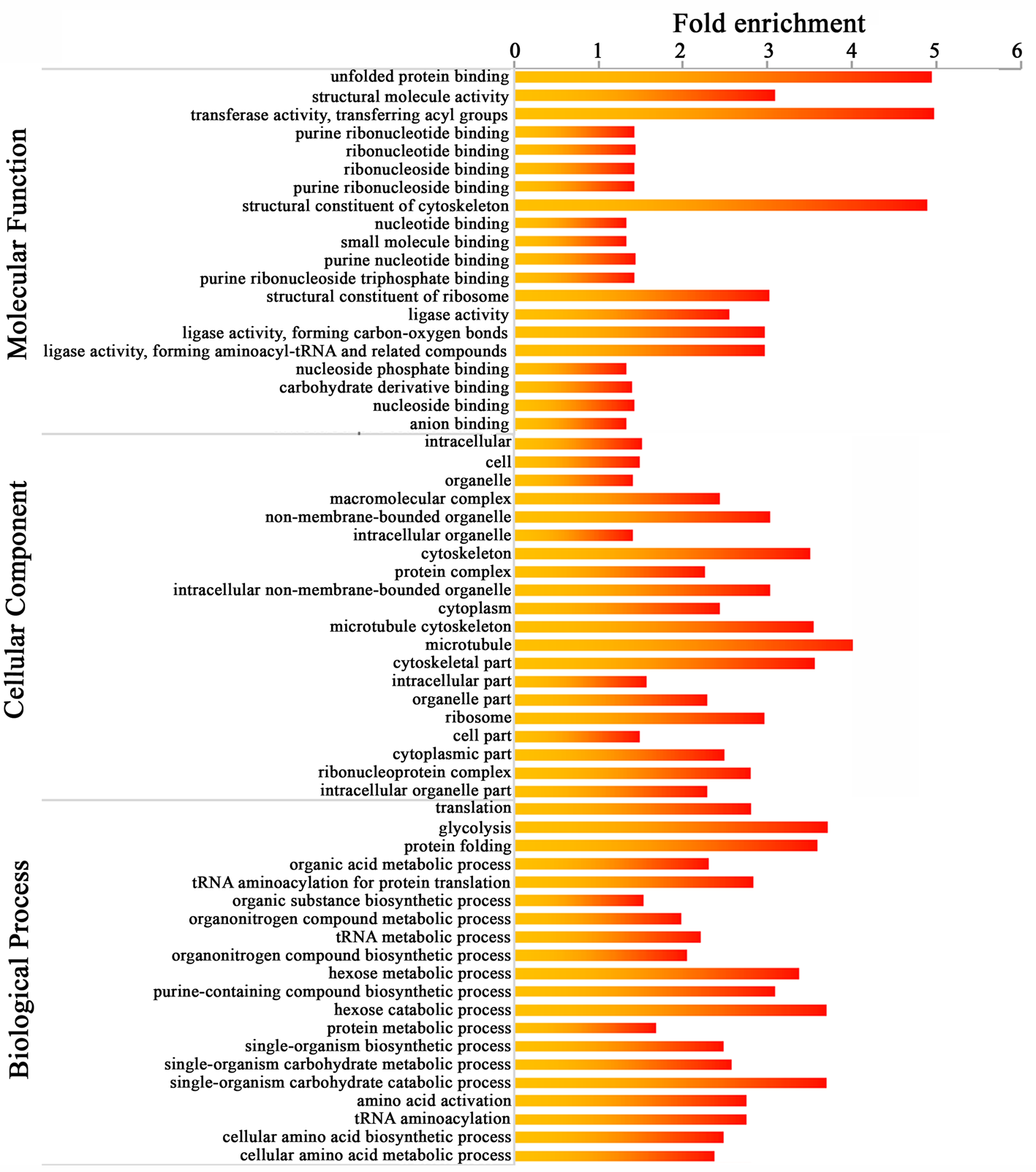


**Figure S2. Protein domain enrichment analysis of identified proteins.**


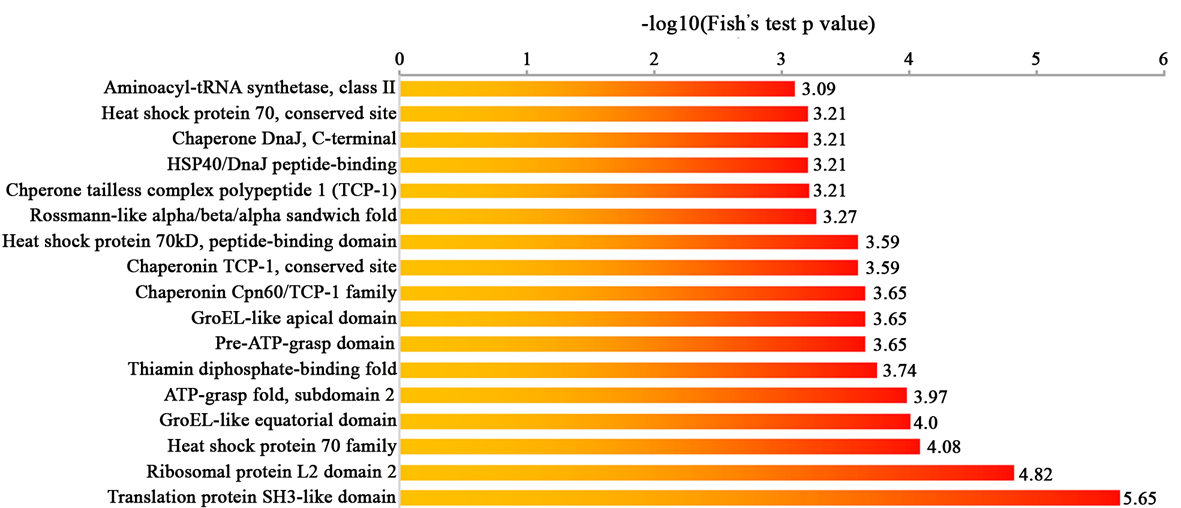


**Figure S3. Protein-protein interaction networks of identified acetylated proteins.**

**
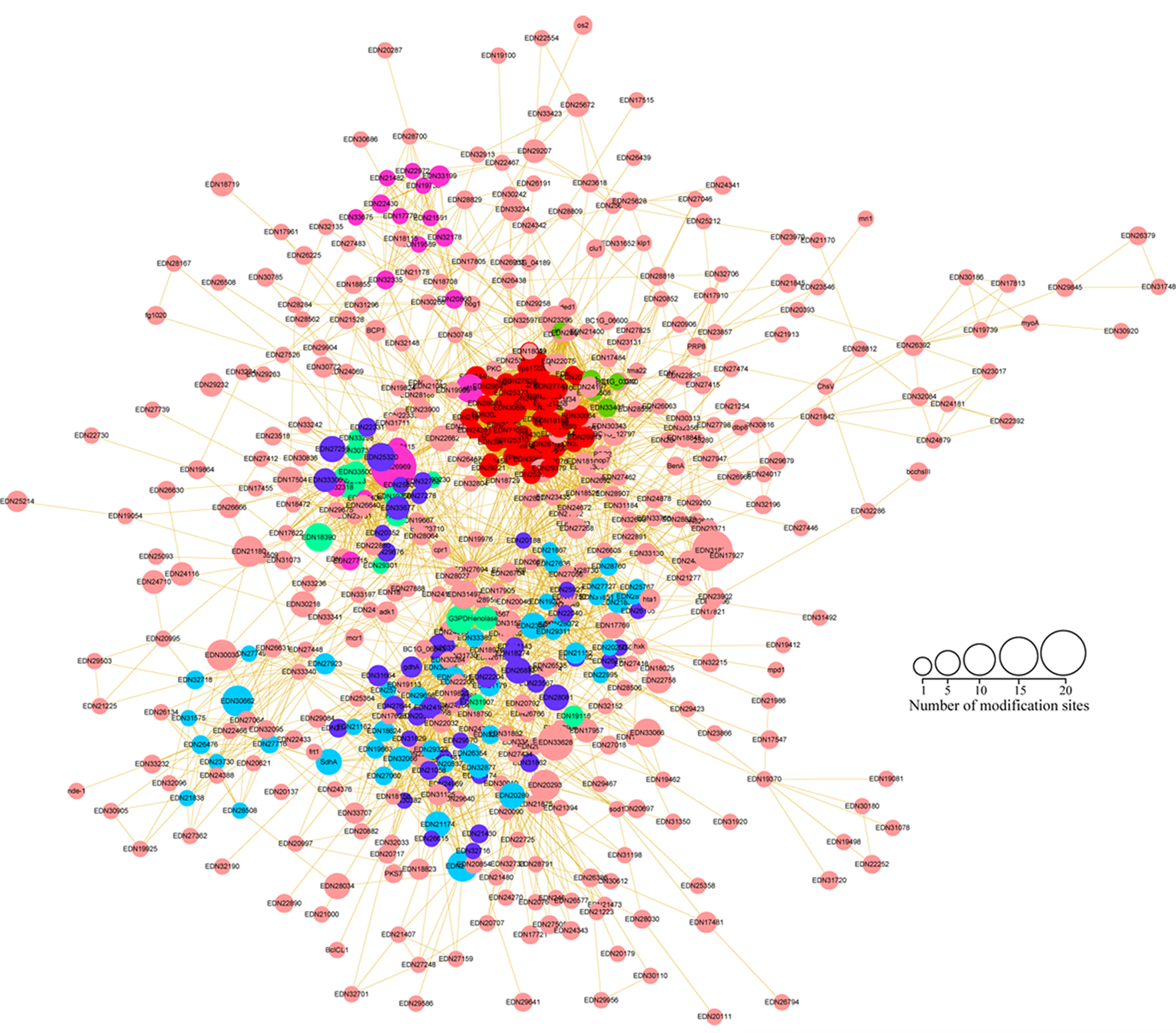
**

**Figure S4. Protein-protein interaction subnetworks of identified acetylated proteins.**


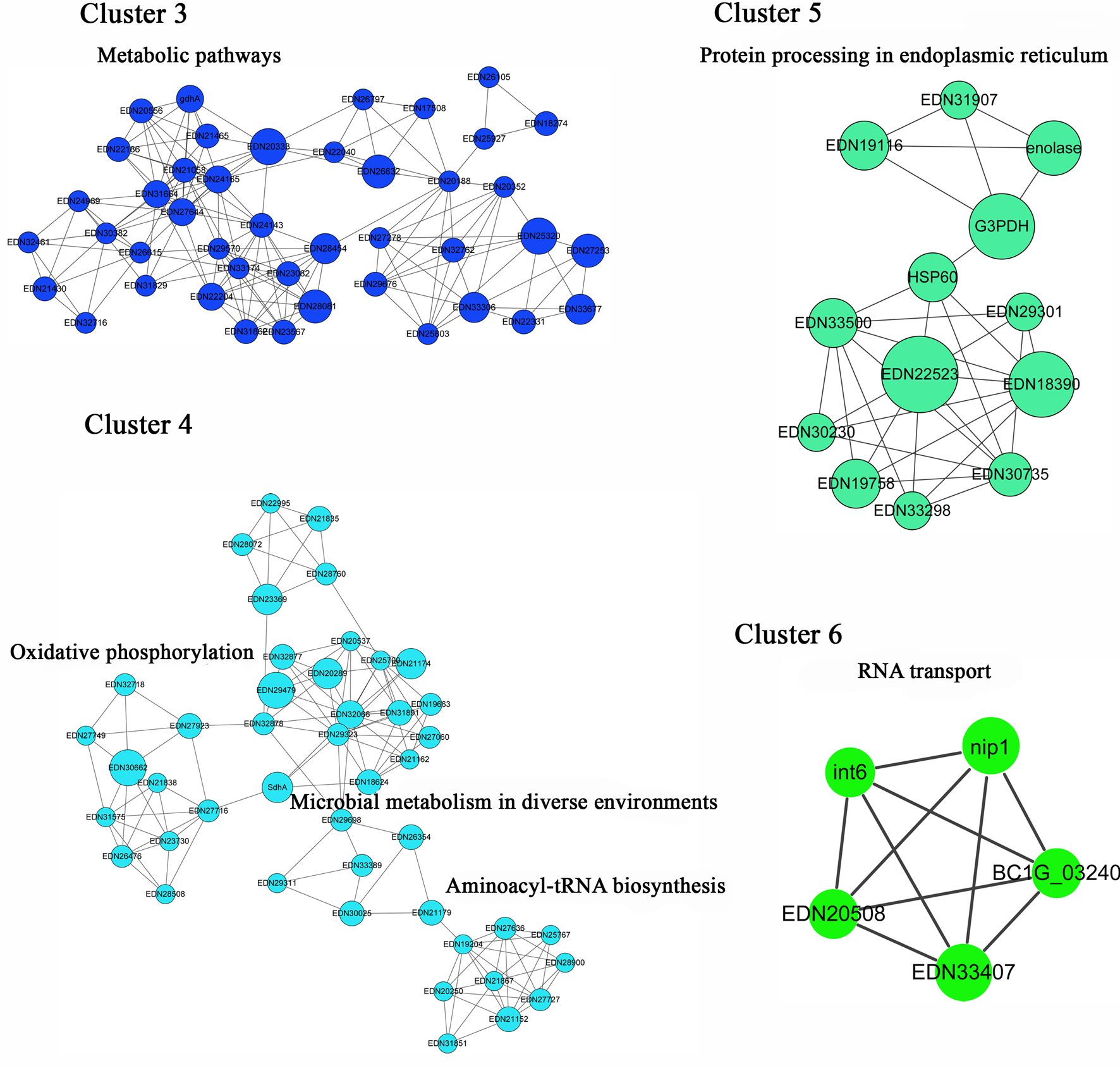

Supplement: Supplementary Figures [file srep29313-s1.doc]
